# Therapeutic Fairytales for Holistic Child Development: A Systematic Literature Review of Clinical, Educational, and Family-Based Practices

**DOI:** 10.34763/jmotherandchild.20242801.d-24-00040

**Published:** 2025-03-21

**Authors:** Andi Sulfana Masri, Agus Nuryatin, Subyantoro Subyantoro, Mukh Doyin, Prusdianto Prusdianto

**Affiliations:** Language Education Doctoral Program, Languages and Literature, Semarang State University, Semarang, Indonesia; Indonesian Language Education Study Program, Languages and Literature, Semarang State University, Semarang, Indonesia; Indonesian Language Education Study Program, Languages and Literature, Semarang State University, Semarang, Indonesia; Indonesian Literature Study Program, Languages and Literature, Semarang State University, Semarang, Indonesia; Performing Arts Study Program, Arts and Design, Makassar State University, Makassar, Indonesia

**Keywords:** Therapeutic Fairytales, Child Development, Clinical, Educational, Family-Based Practices

## Abstract

Therapeutic Fairytales can support child development, and many studies have recognized its benefits for children. However, no one has integrated storytellers as a holistic tool to support children’s overall development, whether in a clinical, educational, or family-based practice context. Therefore, this study conducted a systematic literature review to effectively explain how story therapy can be used in clinical, educational, and family-based practice contexts to support children’s holistic development. The study’s novelty provides a new perspective on story therapy in child development with a systematic approach that highlights the unique contribution of a story as a therapeutic tool that supports mental health, educational aspects, and family dynamics. This systematic literature review follows the PRISMA guidelines to collect 33 articles indexed by the Scopus database identified and selected for review. The review identified two main themes in the context of clinical, educational, and family-based practice: various forms of story therapy utilization and types of child development. The research results show that Therapeutic Fairytales has various benefits in supporting children’s holistic development, including emotional, cognitive, social, moral, and language development. This study implies that Therapeutic Fairytales becomes a more structured and integrated tool in child intervention programs in various fields, inspiring further research on its effectiveness in other contexts.

## Introduction

The holistic development of children is gaining increasing attention, not only in a clinical context but also in the context of integrating educational approaches [1,2,3] and family intervention [4]. Fairy tales have been proven to improve children’s development in various holistic aspects. [5,6]. The influence of fairy tales on children’s development uses various psychological mechanisms [7]. Fairy tales as literature are entertaining, so the various values of goodness in them will be more likely to be understood easily and accepted with a feeling of comfort (happiness) than teaching life values through conventional methods.

The term Therapeutic Fairytales refers to fairytales that are designed or used with the specific purpose of supporting healing, emotional development, and improving the psychological well-being of children [8]. Not all fairytales are directly designed for therapeutic purposes (in the context of psychotherapy). Still, from a broader point of view, every fairy tale can be considered to have therapeutic elements because it always has the nature of Dulce et utile (entertaining and educating). Even in a therapeutic context, the fairytales used are not always Therapeutic Fairytales (in the specific sense); they may also include classic fairytales or folk tales employed as Therapeutic Fairytales in a broader sense [9]. Therefore, this article uses the term “Therapeutic Fairytale” to encompass the entire spectrum of fairytales capable of providing therapeutic effects, whether explicitly designed for therapy or implicitly supporting the holistic development of children. This approach highlights the universal potential of fairytales across various contexts, including educational, clinical, and familial settings.

Recent research has highlighted the importance of preparing teachers to use fairytales in learning as a therapeutic technique for students [9]. This supports the argument that fairy tale interventions can also be categorized as therapeutic actions in non-clinical contexts. In Family-Based Practices, storytelling is done for various purposes [10], such as building emotional bonds and supporting language and literacy development. These are the same as general therapeutic procedures, which are always carried out to positively influence the patient’s development.

In the context of family-based practice, children often ask their parents to read their favourite fairytales repeatedly so that children really remember the details of the story, which has an impact on their development [11]. This is similar to therapy in a special sense, usually done repeatedly. The similarity implies that learning in schools and certain parenting practices at home, which aim to support the development of children, can be seen as a procedure of child development therapy. Thus, fairytales used in therapy in the context of education and family can also be seen as therapeutic fairytales used in therapy to support the development of children.

This study supports previous research on the role of children’s literature in supporting children’s holistic development and well-being [12]. The literature review “Examines pedagogical, didactic and psychological/therapeutic dimensions of children’s literature, to highlight its role in promoting students’ holistic development and well-being” [12]. Previous research did not look at the perspective of utilising fairytales in family-based practices, even though families play an important role in children’s mental health therapy [13] in various areas of child development [14].

In this study, the use of fairy tales is seen in the didactic, clinical, and psychiatric aspects of practice and family-based practices at home. This article offers a new perspective on using fairy tale therapy in child development with a systematic approach that has not been widely discussed in previous literature.

While many studies have recognised the therapeutic benefits of fairy tales, this article will highlight the unique contribution of fairytales as a therapeutic tool that supports mental health, educational aspects, and family dynamics.

By examining clinical, educational, and family-based practice, this article aims to demonstrate that story therapy can be a holistic method to support children’s development. This creates a more comprehensive understanding of how stories can serve as a medium for learning, healing, and strengthening family bonds. Through this systematic literature review, this article contributes to the development of story therapy theory and practice, making it a more accessible and applicable resource for parents and educators in their efforts to support the holistic development of children.

## Methodology

This research uses a Systematic Literature Review of questions formed using systematic and explicit methods to identify, select, and critically evaluate previous research relevant to collecting and analysing data from these studies [15,16]. To conduct a Systematic Literature Review on fairy tale therapy for child development, a search for articles from 2015–2024 was conducted through the Publish or Perish application. The Scopus database was determined to maintain the quality of the articles and for free access in searches published or perished. The keywords used were fairytale, fairy tale, fable, storytelling, storybook, fairytale therapeutic, and fairytale therapy. 672 papers were found consisting of 200 articles each with the following keywords: fairytale storytelling, storybook; 20 articles for fairy tale; 30 articles for fable; 6 for fairytale therapeutic; and 16 for fairytale therapy.

This Systematic Literature Review follows the PRISMA (Preferred Reporting Items for Systematic Review and Meta-Analysis) guidelines [17]. A total of 25 of them were excluded because they were identified as duplicates of the Covidence application. The selected papers were only articles and conference papers, totalling 528 papers. Other papers, such as books/book chapters, reviews, editorials, notes, letters, and short surveys, were excluded. Potentially relevant articles underwent full-text review to determine eligibility for inclusion in our analysis. The inclusion criteria were: 1) articles published in English and French to ensure wide understanding and dissemination in the academic community; 2) articles must mention the use of fairy tales in supporting child development, both in educational (school), clinical (psychotherapy), and family contexts; 3) the article employs Western fairytales or those rooted in Western cultural contexts. Fairytales from different cultural backgrounds possess distinct characteristics [18], and ignoring these differences could result in unwarranted generalisations.

For articles with the keywords storybook and storytelling, at least there is an explanation that the concept of storytelling and storybook in question includes fairytales and does not specifically refer to non-fiction books/stories. Of the 58 relevant articles in English and French, 20 were removed due to closed access, uncertainty of the target group, and uncertainty of fairy tales as the object of research (for example, in articles with the keywords storytelling and storybook, the intended ones are not fairy tales, but non-fiction stories). Finally, 33 articles were left for analysis.

## Result

Data from 33 articles, including title, context (Clinical, Educational, or Family-Based Practices), page, year, author, location, purpose, type of research, research subjects, results, kind of child development targets, and role/utilisation of therapeutic fairytales, were entered into a new list in Excel. Table 1 systematically reviews children’s holistic development from therapeutic fairytale intervention.

**Table 1.** Therapeutic Fairytale on Child Development

**No.**	**Author**	**Location**	**Targets age**	**Therapeutic Fairytale On Child Development**
1.	O. H. Kucheriavyi	Ukraine	Students in grades 1 to 4 of elementary school	Development of understanding of physics & mathematics concepts (Cognitive)
2.	So Jung Kim dan Alyse C. Hachey	South Korea	Children aged five years	Literacy skills (Language) and critical thinking skills (cognitive)
3.	Clara Vidal Carulla, et al.	Sweden	Children aged around three years	Development of executive functions (cognitive)
4.	Gabrielle Katie Spears	England	30 students in grade 3 of elementary school students in junior high school	Development of self-confidence (affective), concept of ownership and understanding (Cognitive)
5.	A. Nicolopoulou, et al.	United States	Preschoolers aged 3 and 4	Development of narrative comprehension (cognitive); language; and attitude development
6.	Enni Vaahtoranta, et al.	West Germany	Preschoolers aged 4 to 6	Language development, focus and anxiety reduction during storytelling sessions
7.	Su-Jeong Wee, et.al	South Korea	Five-year-olds	development of story comprehension, critical thinking, attitude of challenging stereotypes
8.	Sergio Agnoli, et al.	Italy	Children in grades 3 to 5 of elementary school	Creative thinking development
9.	Sarah A. Brown, et.al	United States	2nd and 3rd graders	Development of understanding the concept of natural selection
10.	Seung-Hee Claire Son, et al.	United States	Kindergarten and 2nd graders	development of understanding the story
11.	Avid K. Dickinson, et al.	United States	Preschoolers with an average age of 57.3 months	development of language (vocabulary)
12.	Leydi Johana Chaparro-Moreno, et.al	Colombia	Preschoolers with an average age of 43 to 55 months	language development
13.	Gloria Yi-Ming Kao, et al.	Taiwan	Fourth graders	Development of reading motivation, story comprehension and chromatic concept
14.	Brenna Hassinger-Das, et al.	United States	Kindergarten children with early numeracy difficulties	Development of mathematical vocabulary understanding
15.	Elizabeth Spencer Kelley, et al.	United States	The average age of preschool children is 4 years 6 months	Development of story understanding
16.	Natalie Emmons, et al.	United States	Kindergarten and second grade children from two elementary schools	Development of understanding of the concept of natural selection
17.	Caren M. Walker, et al.	United States	Preschool children ages 3, 4, and 5 years	Development of story understanding
18.	Ninger Zhou dan Aman Yadav.	United States	The average age of preschool children is 4.4 years	Development of children’s involvement in reading) language development (vocabulary)
19.	John J. Heilmann, et al.	United States	Children in kindergarten through third grade	Language development
20.	Manyande A, et al.	Belgium, France, Italy, Portugal, Spain, and United States	Children under 18 years of age	Psychological development (Anxiety reduction)
21.	A. Jaeger, et al.	France	12 year old Margaret (non-pubescent)	Psychological development
22.	Laurel M. Silber	United States	4 year old and 9 year old boys	Development of coping mechanisms and mother-child attachment
23.	Ann-Marie Jelena Golden, Miro	Croatia	Boys aged 4 and 9 years old	Psychological development (reduction in symptomatology, disappointment and healing)
24.	Stefania La Foresta, et al.	Italy	Children aged between 2 months to 14 years	Psychological development (anxiety reduction)
25.	Yusuf Akemoglu dan Kimberly R. Tomeny	United States	The children were between 38 and 71 months old	Communication (language) development
26.	Natalia Kucirkova	England and Norway	Early childhood	Cognitive empathy development.
27.	Lorna G. Hamilton, et al.	England	Children at risk for dyslexia and those without, aged 3.5 to 9 years	Language development and reading skills
28.	Michelle I. Brown, et al.	Australia	Babies between 3 to 12 months	Development of language and social communication skills
29.	Marina L. Puglisi, et al.	England	Children The average age of children is 3.5 years, who are at high risk of developing dyslexia.	Children’s language development and reading/spelling skills
30.	Pamela W. Burris, et al.	United States	Preschool children from low-income families.	Receptive and expressive language development
31.	Lorenz Grolig	Berlin, Germany	Early childhood in the home environment and child care settings.	Oral language development
32.	Lorenz Grolig, et al.	Germany	Preschool children with an average age of 5 years 5 months	Language development
33.	Pooja Pandith, et al.	India	Preschool children aged 3 to 5 years	Development of interest in reading story books

All articles that met the inclusion criteria touched on the role of fairytales in the development of children between the ages of 2 months and 17 years. This systematic review found that therapeutic fairytales in the context of clinical, educational, and family-based practices play a role in the holistic development of children.

In a clinical context, therapeutic fairytales play a role in language development [19]; cooperation during medical procedures [20]; emotional and psychological development, including anxiety reduction, healing from trauma [20,21,22,23], development of coping mechanisms and mother-child attachment [22].

In the clinical context of psychotherapy, storytelling plays a role in healing certain traumatic impacts. Therapy using storytelling is personalised to promote psychological growth, meaning in life, resilience, self-realisation, and increased well-being [21,23,24]. Fairytales are used as metaphors to describe the condition of children who experience trauma. One article metaphors the traumatic condition of children in the fairy tale Humpty Dumpty [22].

In the educational context, Fairy tales play a role in the development of narrative understanding [25,26,27,28,29] and conceptual understanding [27,30,31,32]. The development of critical thinking includes questioning, analysing, and exploring different perspectives from the fairy tales they read [33,34]. The development of creative thinking includes Divergent Thinking (the ability to generate many alternative ideas or different solutions to a problem) and Integrative Thinking (the ability to combine various elements into one original and effective idea) [35]. Executive function development includes Inhibitory Control/IC (the ability to control impulses and focus), Working Memory/WM (the ability to remember instructions and information), and Attention Shifting/AS (the ability to switch from one perspective or task to another) [36]. Imagination development is obtained from the ability to create fairy tales [25]. Language development includes the development of grammatical skills (phonology, morphology, vocabulary, and sentences) [25,33,37,38,39,40], narrative language [25], and science vocabulary [40]. The development of social skills through learning fairytales, including interaction skills and self-regulation in social situations [25]. The development of students’ moral values in learning through fairy tales cannot be denied because fairy tales contain various moral and character values, such as religiosity, hard work, creativity, discipline, and responsibility [34], also including self-confidence [31].

In the context of Family-Based Practices, therapeutic fairytales play a role in language, emotional, and social development domains. Fairytales play a role in language development, including grammatical language development to narrative understanding [41,42,43,44,45]. Fairytales play a role in children’s emotional development, especially empathy [46]. Fairytales play a role in social development, especially the skills of initiating and maintaining communication [42,47].

Children’s exposure to storybooks (especially fairy tales) contributes significantly to Lower Level Language Skills (LLL) and Higher Level Language Skills (HLL) [41,42,43,44,45, 47,48,49]. Children also showed receptive language development (understanding words heard) and expressive language (using words to express thoughts and feelings) through home storytelling interventions [44,47]. Storytelling not only mediates the development of reading skills [43] but also speaks [48]. Storytelling not only mediates the development of verbal skills but also non-verbal (eye contact, joint attention, and use of gestures) through responses to questions and instructions during reading sessions [47]. Storytelling activities together support children’s development in initiating communication [42,47]. It should be noted that appropriate reading techniques support the development of language skills through reading fairy tales or support children’s involvement [47], the intensity of reading exposure [44,49] and parental awareness of the importance of reading fairytales [49]. Fairytales are also said to play a role in the development of children’s empathy [46]. Fairytales play a role in children’s social development [42].

## Discussion

In general, this study found that fairy tales used in clinical, educational, and Family-Based Practices contexts have therapeutic qualities that support children’s holistic development. The fairy tales employed to support children’s development are not limited to those specifically created for developmental and therapeutic purposes but also include general fairy tales such as classic tales like *The Little Mermaid* or *Peter Pan* [50]. These findings further reinforce the use of the term *therapeutic fairytale* to describe the general potential of fairy tales as a medium for supporting holistic development. These findings and opinions are supported by other research, which also found that traditional fairy tales not specifically designed for healing purposes can be utilised for therapeutic aims and have been proven to have therapeutic effects on well-being and positive development, particularly among stressed women [51].

In the clinical context, fairy tales and their application procedures are selected using an individualised approach tailored to each child’s specific needs to support their language, social, emotional, and psychological development.

This is because fairy tale interventions aim to facilitate healing, whether addressing language issues, social development challenges, or emotional and psychological concerns.

Regarding emotional and psychological development, fairy tales with symbolism that aligns with the patient’s condition and their appropriate use are effective in addressing and healing psychological issues such as anxiety, depression, excessive fear, and low self-esteem, often rooted in traumatic experiences or loss [21,22,24,52]. Children with trauma frequently struggle to integrate their emotional experiences. Fairy tales, with their symbolic nature, enable children to project their emotions onto characters or situations within the story. For instance, children may project feelings of being “broken” through the symbolism of the fairy tale *Humpty Dumpty* [22]. Fairy tale interventions, such as reading or completing stories relevant to a child’s psychological challenges, help them understand and accept their emotions. Writing fairy tales provides a safe space for children to express their feelings without fear of judgment or threat. These interventions can also be combined with play or drawing therapy as a means for children to present and express their emotions [22]. This allows therapists to gain deeper insights into the patient’s emotional and psychological state, which is crucial for the healing process.

One of the language development issues that can be addressed through fairy tale interventions is the narrative language development of bilingual children [19]. This includes limitations in lexical diversity, mean length of utterance, narrative structure, and coherence in story construction. Interventions using wordless picture storybooks are particularly relevant, as fairy tales contain words, phrases, sentences, and paragraphs organised into cohesive stories. By practising storytelling based on wordless picture storybooks, children enhance their ability to construct words, form sentences, and organise ideas logically into engaging and coherent narratives. This process develops vocabulary sentence structure and broader narrative skills, such as connecting events, describing characters, and conveying conflict and resolution verbally. Thus, fairy tales serve as an effective tool for supporting holistic language development [19].

In the context of social and emotional development, engaging fairy tales in visual formats, such as a child’s favourite video fairy tale, can help them cooperate more easily with family and medical staff during clinical procedures [20,23]. Watching or listening to their favourite fairy tales distracts children from the intimidating clinical environment and reduces their anxiety. This makes children more cooperative as they feel safer and more comfortable, fostering a positive social atmosphere between the child, parents, and medical staff. It also provides children with an experience of managing emotions to create positive social interactions, which they can replicate in other social contexts. Furthermore, the success of fairy tale interventions during medical procedures, such as anaesthesia induction or Intrathecal Nusinersen, significantly impacts the patient’s physical recovery and overall holistic development.

In the educational context, therapeutic storytelling supports children’s language and literacy, as well as cognitive, social, and moral development. To achieve this goal, storytelling is implemented in group-based learning practices. Unlike clinical contexts, which use a personalised approach, the school environment emphasises collective needs while still considering individual requirements and their relevance to everyday life, such as the general needs of early childhood education (ECE) students. Attention to individual needs ensures that each child can be actively involved and benefit optimally from learning activities. For children undergoing trauma recovery, this approach allows the therapeutic effects of storytelling to work subtly on the subconscious level, as storytelling is not explicitly designed for healing. Thus, storytelling promotes the holistic development of children through inclusive, relevant, and enjoyable learning experiences.

In relation to the aspect of language development, wordless storybooks are also used in educational contexts to stimulate narrative production, as in clinical contexts. Story interventions in educational contexts are not only to support narrative language development but also to enrich vocabulary in specific areas, such as mathematics and science vocabulary [30,40]. This supports holistic language development. In addition, technology-based story interventions are also used to support children’s language development, for example, by using digital story books (e-books) equipped with animation [26]. This opens opportunities for children to develop not only their language skills but also other skills, such as cognitive, imagination, or digital literacy through educational contexts.

Fairy tales uniquely support children’s cognitive development. Through stories, children learn to follow plots, understand cause-and-effect relationships, and intuitively grasp moral lessons. Critical thinking develops as they analyse characters’ actions and seek solutions, while creative thinking is stimulated by creating new endings or retelling stories from different perspectives. Executive functions, such as working memory and impulse control, are enhanced by recalling story details and engaging in role-play. Additionally, their imagination expands through exploring the fantastical world of fairy tales, fostering creativity. With this approach, fairy tales serve as an effective tool for promoting holistic cognitive development.

As explained in the clinical context, fairy tale interventions in education are also combined with play therapy, such as the Storytelling and Story-Acting (STSA) method, which is effective for children’s social development [25]. This activity enhances communication skills, teamwork abilities, and self-regulation, including impulse control and the capacity to share roles and responsibilities. Research [53] supports that learning should employ various techniques to foster children’s development, including simple storytelling followed by fairy tale dramatisation involving painting, construction, and exercises. Combining fairy tale interventions with play therapy can be a valuable approach to maximising the therapeutic effects of fairy tales on children’s development.

Fairytales play a crucial role in shaping children’s morals by teaching ethical values and character, such as distinguishing between right and wrong and understanding the story’s message. The selection of fairytales should consider cultural contexts, as each culture has different value priorities. For example, in Indonesia [53], fairytales instil values related to relationships with God, oneself, family, society, and the environment, while in Western cultures, they tend to emphasise individualism or freedom of expression. Thus, fairytales serve as an effective medium for conveying moral values tailored to the cultural needs of each society.

Therapeutic fairy tales play a crucial role in supporting children’s language, emotional, cognitive, and social development in the context of family-based caregiving. Fairy tales in the family setting are particularly important for fostering development through activities such as reading aloud or storytelling. Reading fairy tales effectively promotes children’s growth [46]. The selection of stories, reading methods, and the repetition or intensity of reading activities significantly enhance the effectiveness of these activities in supporting children’s development.

Interactive shared reading, which emphasises child engagement while maintaining contact and intimacy between parents and children, should be considered. This method not only supports language and cognitive development but also strengthens parent-child attachment (emotional development) [41] and enhances communication and empathy skills (social development) [18,21]. Conducted in a relaxed home setting, parents often ask simple questions or provide comments to expand the story, creating opportunities for dialogue, reflection, and the reinforcement of social skills in a comfortable and enjoyable environment.

The limited time allocated for fairy tale therapy in schools and clinical contexts often restricts its full potential. In this regard, the home environment is crucial to filling these gaps. Families, particularly parents, are responsible for complementing and reinforcing the learning provided at school or through clinical therapy. By engaging children in regular fairy tale reading activities at home, parents can amplify the positive effects of therapy, enrich their learning experiences, and create an environment that supports comprehensive cognitive, emotional, and social development. Collaboration between home, school, and clinical therapy is essential to ensure continuity and maximise the impact of learning.

In the context of cognitive development, fairy tales in family settings focus more on laying the foundation for early development. They informally build cognitive basics, such as enriching vocabulary, improving memory, and enhancing narrative comprehension. This contrasts with the educational context, where fairy tales are utilised to hone higher-order and complex thinking skills, such as critical thinking, abstract concept understanding, executive functions, and logic. Thus, fairy tales in family settings serve as an informal foundation for cognitive development, while in education, they target advanced thinking skills. Together, they complement each other in supporting a child’s comprehensive cognitive growth. This systematic review provides evidence that the family environment also plays a crucial role in child development, suggesting that interventions typically applied in schools and clinical practices, such as therapeutic fairy tales, should also be implemented at home. The parent-child relationship has the greatest impact among the many relationships influencing a child’s development. Responsive parenting is a key factor in promoting early childhood development [54]. Across various countries, cultures, and contexts, caregiver support programs have improved short-term Early Childhood Development (ECD) outcomes by fostering responsive parenting and age-appropriate caregiver-child interactions [55]. Recent efforts to extend the relational health model into the field of child development emphasise the roles of parents, children, and contextual factors in supporting and maintaining healthy parent-child relationships [56].

## Conclusions

Beyond clinical and educational contexts, family-based fairy tale interventions are vital in supporting children’s holistic development. Families can complement clinical practices and fairy tale-based learning by replicating practices such as shared reading at home and establishing reading routines. With active family involvement, fairy tale interventions in all contexts can profoundly impact holistic child development.

These findings guide healthcare and therapy practitioners, educators, and families to design specific, targeted, and evidence-based fairy tale interventions. Collaboration among schools, families, and clinical professionals is also crucial to maximising the benefits of fairy tales as a developmental intervention. Adopting approaches tailored to the child’s needs and environment, fairy tales can serve as a dynamic and relevant tool to support their comprehensive growth.

## References

[1] Condello G, Mazzoli E, Masci I, De Fano A, Ben-Soussan TD, Marchetti R (2021). Fostering holistic development with a designed multisport intervention in physical education: A class-randomized cross-over trial. Int J Environ Res Public Health.

[2] Carter D (2016). A nature-based social-emotional approach to supporting young children’s holistic development in classrooms with and without walls: The social-emotional and environmental education development (SEED) framework. Int J Early Child Environ Educ.

[3] O’Flaherty J, McCormack O (2019). Student holistic development and the ‘goodwill’ of the teacher. Educ Res.

[4] Olalowo I, Babalola A (2024). A tale of two childhoods: exploring holistic development of preschoolers in a rural and urban community. Theory Pract Child Dev.

[5] Kulikovskaya IE, Andrienko AA (2016). Fairy-tales for modern gifted preschoolers: developing creativity, moral values and coherent world outlook. Procedia - Soc Behav Sci.

[6] Hibbin R (2016). The psychosocial benefits of oral storytelling in school: developing identity and empathy through narrative. Pastor Care Educ.

[7] Kuciapiński MJ (2014). The Therapeutic and Educational Properties of Fairytale Therapy in the Early Stages of Children’s Development. Pedagogika Rodziny. Family Pedagogy.

[8] Jones P, Pimenta S (2020). The storm: for children growing through parents’ separation.

[9] Aigul A, Feruza Y, Onal A, Alena G, Sandugash K, Aktoty K (2024). Preparing future teachers and psychologists to use integrative fairy tale therapy techniques. Eurasian J Appl Linguist.

[10] Vasalou A, Kalantari S, Kucirkova N, Vezzoli Y (2020). Designing for oral storytelling practices at home: A parental perspective. Int J Child-Computer Interact.

[11] Hailey DJ, Hailey TI, Fazio-Brunson M (2019). Family stories: a teaching tool for families and early childhood educators. Int J Humanit Soc Sci.

[12] Pulimeno M, Piscitelli P, Colazzo S (2020). Children’s literature to promote students’ global development and wellbeing. Health Promot Perspect.

[13] Cobham VE, McDermott B, Haslam D, Sanders MR (2016). The role of parents, parenting and the family environment in children’s post-disaster mental health. Curr Psychiatry Rep.

[14] Boerma IE, Mol SE, Jolles J (2017). The role of home literacy environment, mentalizing, expressive verbal ability, and print exposure in third and fourth graders’ reading comprehension. Sci Stud Read.

[15] Yusoff MJHM, Sulaiman MZ, Zainudin IS, Haroon H (2023). Sistem pentauliahan penterjemah: sorotan literatur bersistematik [Translator certification system: a systematic literature review]. GEMA Online® J Lang Stud.

[16] Ismail MS, Sarudin A (2023). Sorotan literatur bersistematik: interpretasi partikel wacana “lah” dalam ujaran masyarakat Malaysia [Systematic literature review: interpretation of “lah” discourse particles in Malaysian speech]. GEMA Online® J Lang Stud.

[17] Riedmeier M, Decarolis B, Haubitz I, Müller S, Uttinger K, Börner K (2021). Adrenocortical carcinoma in childhood: A systematic review. Cancers (Basel).

[18] Eslit ER (2023). Tales through a cultural lens: exploring the global significance of Cinderella, Snow White, and Sleeping Beauty. Int J Lang Cult.

[19] Heilmann JJ, Rojas R, Iglesias A, Miller JF (2016). Clinical impact of wordless picture storybooks on bilingual narrative language production: A comparison of the “frog” stories. Int J Lang Commun Disord.

[20] Manyande A, Cyna AM, Yip P, Chooi C, Middleton P (2015). Nonpharmacological interventions for assisting the induction of anaesthesia in children. Cochrane Database Syst Rev.

[21] Jaeger A, Bofferding C, Prudent C, Vervier JF, de Tychey C (2017). Clinique d’une enfant endeuillée par le suicide de son père : identification mélancolique [Suicide attempt in childhood and melancholic identification: A clinical projective approach]. Neuropsychiatr Enfance Adolesc.

[22] Silber LM (2020). Reimagining Humpty Dumpty with play’s therapeutic action. J Infant, Child, Adolesc Psychother.

[23] Golden A-MJ, Hančević Horvat N, Jakovljević M (2021). Acceptance and change as dialectic of recovery: examples of storytelling, fairy tale and psychopharmacotherapy as therapeutic modalities. Psychiatr Danub.

[24] La Foresta S, Faraone C, Sframeli M, Vita GL, Russo M, Profazio C (2018). Intrathecal administration of Nusinersen in type 1 SMA: successful psychological program in a single Italian center. Neurol Sci.

[25] Nicolopoulou A, Cortina KS, Ilgaz H, Cates CB, de Sá AB (2015). Using a narrative- and play-based activity to promote low-income preschoolers’ oral language, emergent literacy, and social competence. Early Child Res Q.

[26] Son SHC, Butcher KR, Liang LA (2020). The influence of interactive features in storybook apps on children’s reading comprehension and story enjoyment. Elem Sch J.

[27] Kao GYM, Tsai CC, Liu CY, Yang CH (2016). The effects of high/low interactive electronic storybooks on elementary school students’ reading motivation, story comprehension and chromatics concepts. Comput Educ.

[28] Kelley ES, Goldstein H, Spencer TD, Sherman A (2015). Effects of automated Tier 2 storybook intervention on vocabulary and comprehension learning in preschool children with limited oral language skills. Early Child Res Q.

[29] Walker CM, Gopnik A, Ganea PA (2015). Learning to learn from stories: children’s developing sensitivity to the causal structure of fictional worlds. Child Dev.

[30] Kucheriavyi OH (2022). Didactic fairy tale designing as a key to proactive training of Physics and Mathematics at primary schools. J Phys: Conf Ser.

[31] Spears GK (2021). Breaking the gender binary: using fairytales to transform playground possibilities for year 3 girls. Educ 3–13.

[32] Brown SA, Ronfard S, Kelemen D (2020). Teaching natural selection in early elementary classrooms: can a storybook intervention reduce teleological misunderstandings?. Evol Educ Outreach.

[33] Kim SJ, Hachey AC (2021). Engaging preschoolers with critical literacy through counter-storytelling: a qualitative case study. Early Child Educ J.

[34] Wee SJ, Kim KJ, Lee Y (2019). ‘Cinderella did not speak up’: critical literacy approach using folk/fairy tales and their parodies in an early childhood classroom. Early Child Dev Care.

[35] Agnoli S, Pozzoli T, Mancini G, Franchin L, Mastria S, Corazza GE (2022). This is my fairy tale: how emotional intelligence interacts with a training intervention in enhancing children’s creative potential. J Creat Behav.

[36] Vidal CC, Christodoulakis N, Adbo K (2021). Development of preschool children’s executive functions throughout a play-based learning approach that embeds science concepts. Int J Environ Res Public Health.

[37] Vaahtoranta E, Suggate S, Jachmann C, Lenhart J, Lenhard W (2018). Can explaining less be more? Enhancing vocabulary through explicit versus elaborative storytelling. First Lang.

[38] Dickinson DK, Nesbitt KT, Collins MF, Hadley EB, Newman K, Rivera BL (2019). Teaching for breadth and depth of vocabulary knowledge: Learning from explicit and implicit instruction and the storybook texts. Early Child Res Q.

[39] Chaparro-Moreno LJ, Reali F, Maldonado-Carreño C (2017). Wordless picture books boost preschoolers’ language production during shared reading. Early Child Res Q.

[40] Hassinger-Das B, Jordan NC, Dyson N (2015). Reading stories to learn math: Mathematics vocabulary instruction for children with early numeracy difficulties. Elem Sch J.

[41] Hamilton LG, Hayiou-Thomas ME, Hulme C, Snowling MJ (2016). The home literacy environment as a predictor of the early literacy development of children at family-risk of dyslexia. Sci Stud Read.

[42] Brown MI, Westerveld MF, Trembath D, Gillon GT (2018). Promoting language and social communication development in babies through an early storybook reading intervention. Int J Speech Lang Pathol.

[43] Puglisi ML, Hulme C, Hamilton LG, Snowling MJ (2017). The home literacy environment is a correlate, but perhaps not a cause, of variations in children’s language and literacy development. Sci Stud Read.

[44] Burris PW, Phillips BM, Lonigan CJ (2019). Examining the relations of the home literacy environments of families of low SES with children’s early literacy skills. J Educ Stud Placed Risk.

[45] Grolig L, Cohrdes C, Tiffin-Richards SP, Schroeder S (2019). Effects of preschoolers’ storybook exposure and literacy environments on lower level and higher level language skills. Read Writ.

[46] Kucirkova N (2019). How could children’s storybooks promote empathy? A conceptual framework based on developmental psychology and literary theory. Front Psychol.

[47] Akemoglu Y, Tomeny KR (2021). A parent-implemented shared-reading intervention to promote communication skills of preschoolers with autism spectrum disorder. J Autism Dev Disord.

[48] Grolig L (2020). Shared storybook reading and oral language development: A bioecological perspective. Front Psychol.

[49] Pandith P, John S, Bellon-Harn ML, Manchaiah V (2022). Parental perspectives on storybook reading in Indian home contexts. Early Child Educ J.

[50] Golden A-MJ, Hančević Horvat N, Jakovljević M (2021). Acceptance and change as dialectic of recovery: examples of storytelling, fairy tale and psychopharmacotherapy as therapeutic modalities. Psychiatr Danub.

[51] Ruini C, Masoni L, Ottolini F, Ferrari S (2014). Positive narrative group psychotherapy: the use of traditional fairy tales to enhance psychological well-being and growth. Psychol Well Being.

[52] Athiroh WS, Ahmad R (2021). Relevansi dongeng dengan membentuk karakter anak usia dini [The relevance of fairy tales in forming the character of early childhood]. Golden Age: J Pendidik Anak Usia Dini [Internet].

[53] Sotiropoulou E, Kasapi C (2022). Fairy tale as a pedagogical tool for children under the age of 3: educators’ views and practices. Glob J Educ Stud.

[54] Pitchik HO, Tofail F, Rahman M, Akter F, Sultana J, Shoab AK (2021). A holistic approach to promoting early child development: A cluster randomised trial of a group-based, multicomponent intervention in rural Bangladesh. BMJ Glob Heal.

[55] Aboud FE, Yousafzai AK (2015). Global health and development in early childhood. Annu Rev Psychol.

[56] Frosch CA, Schoppe-Sullivan SJ, O’Banion DD (2019). Parenting and child development: a relational health perspective. Am J Lifestyle Med.

